# COVID-19 Severe acute respiratory syndrome coronavirus 2 (SARS-CoV-2) infection in children and adolescents: a systematic review of critically unwell children and the association with underlying comorbidities

**DOI:** 10.1007/s00431-020-03801-6

**Published:** 2020-09-10

**Authors:** Nia Williams, Trisha Radia, Katharine Harman, Pankaj Agrawal, James Cook, Atul Gupta

**Affiliations:** 1 Department of Paediatric Medicine, King’s College Hospital, London, UK; 2 Department of Paediatric Respiratory Medicine, King’s College Hospital, London, SE5 9RS UK; 3 Institute for Women’s and Children’s Health, King’s College London, London, UK

**Keywords:** Paediatric, Adolescent, COVID-19, Severe acute respiratory syndrome coronavirus 2 (SARS-CoV-2), Critically unwell, Comorbidities

## Abstract

Data show that children are less severely affected with SARS-Covid-19 than adults; however, there have been a small proportion of children who have been critically unwell. In this systematic review, we aimed to identify and describe which underlying comorbidities may be associated with severe SARS-CoV-2 disease and death. The study protocol was in keeping with Preferred Reporting Items for Systematic Reviews and Meta-analyses (PRISMA) guidelines. A total of 1726 articles were identified of which 28 studies fulfilled the inclusion criteria. The 28 studies included 5686 participants with confirmed SARS-CoV-2 infection ranging from mild to severe disease. We focused on the 108 patients who suffered from severe/critical illness requiring ventilation, which included 17 deaths. Of the 108 children who were ventilated, the medical history was available for 48 patients. Thirty-six of the 48 patients (75%) had documented comorbidities of which 11/48 (23%) had pre-existing cardiac disease. Of the 17 patients who died, the past medical history was reported in 12 cases. Of those, 8/12 (75%) had comorbidities.

*Conclusion:* Whilst only a small number of children suffer from COVID-19 disease compared to adults, children with comorbidities, particularly pre-existing cardiac conditions, represent a large proportion of those that became critically unwell.

**Table:** 

**What is Known:** *• Children are less severely affected by SARS-CoV-2 than adults.* *• There are reports of children becoming critically unwell with SARS-CoV-2 and requiring intensive care.*	
**What is New:** *• The majority of children who required ventilation for SARS-CoV-2 infection had underlying comorbidities.* *• The commonest category of comorbidity in these patients was underlying cardiac disease.*

**What is Known:**

*• Children are less severely affected by SARS-CoV-2 than adults.*

*• There are reports of children becoming critically unwell with SARS-CoV-2 and requiring intensive care.*

**What is New:**

*• The majority of children who required ventilation for SARS-CoV-2 infection had underlying comorbidities.*

*• The commonest category of comorbidity in these patients was underlying cardiac disease.*

## Introduction

A cluster of cases of pneumonia of unknown cause was reported in Wuhan, China in December 2019 leading to the identification of a novel severe acute respiratory syndrome coronavirus 2 (SARS-Co-V-2) [1]. The virus has spread rapidly, causing wide outbreaks of the associated disease coronavirus disease 2019 (COVID-19) throughout the globe [2].

In adults, the spectrum of disease is well described ranging in severity from asymptomatic carriage to respiratory failure and death [3]. Following the diagnosis of the first paediatric patient with SARS-CoV-2 in China on January 20, 2020, children across the world have been infected [4]. Data show that children are less severely affected than adults, representing approximately 5% of those infected and less than 1% of hospital admissions [5, 6]. However; there have been a small proportion of children who have been critically unwell requiring intensive care with reported fatalities in children under the age of 18. A recent systemic review assessing the clinical features and management of children with SARS-CoV-2 infection reported that children were most likely to have mild symptoms, predominantly respiratory with a minority reporting gastrointestinal symptoms. Only 1 patient was identified with severe disease requiring intensive care, and no data was available on the role of comorbidities in the severity of paediatric COVID-19 [7].

There is a dearth of studies describing an association between risk factors and comorbidities and severe SARS-CoV-2 disease in children. This is relevant; as we move to the recovery phase of the pandemic and shielding restrictions are being relaxed, it is vital to identify patients who are at high risk of severe disease to be able to advise appropriately. In this systematic review, we aimed to identify and describe which underlying comorbidities may be associated with severe SARS-CoV-2 disease and death in children.

## Methods

### Search strategy and data sources

To identify studies reporting clinical features of children who were critically unwell, defined as requiring invasive mechanical ventilation, with laboratory confirmed SARS-CoV-2 infection, we systematically searched the MEDLINE (PubMed) electronic database from December 1st 2019 to 31st May 2020 using key terms “covid 19 OR coronavirus OR sars-cov2 AND children OR adolescents OR neonate OR infant”. We hand searched the reference lists from retrieved studies and reviews to find additional studies and contacted experts in the field. We also included some additional studies from June 2020 that were found through reading research bulletins so as to not miss key data in this rapidly developing research area.

### Study selection

Two reviewers independently screened titles and abstracts of all citations for eligibility and retrieved those that met the inclusion criteria. If insufficient information was available in the abstract to decide on eligibility, the whole article was retrieved for review. Discrepancies were resolved by discussion and by involving a third reviewer when necessary.

We included case reports, case series, and other observational studies in children under the age of 18. We excluded studies for which the full article was not available and studies that did not contain any original data such as review articles, commentaries and correspondence. Papers reporting information on both children and adults were included only if paediatric data could be retrieved. We excluded studies that focused on other serotypes of severe acute respiratory syndrome coronavirus and Middle East respiratory syndrome coronavirus infection. We excluded papers presenting cases of paediatric multisystem inflammatory syndrome temporally associated with COVID-19 (PIMS-TA).

The study protocol was in keeping with Preferred Reporting Items for Systematic Reviews and Meta-analyses (PRISMA) guidelines.

### Data extraction

A structured data extraction form was piloted and then used to extract data for all included studies by two reviewers in duplicate. For all articles that we included, if available, we extracted the following data: first author, title, year of publication, country, study design, number of cases, gender, age of patients, comorbidities, clinical manifestations, laboratory tests, radiological findings, treatments and outcomes.

## Results

**Fig. 1:**
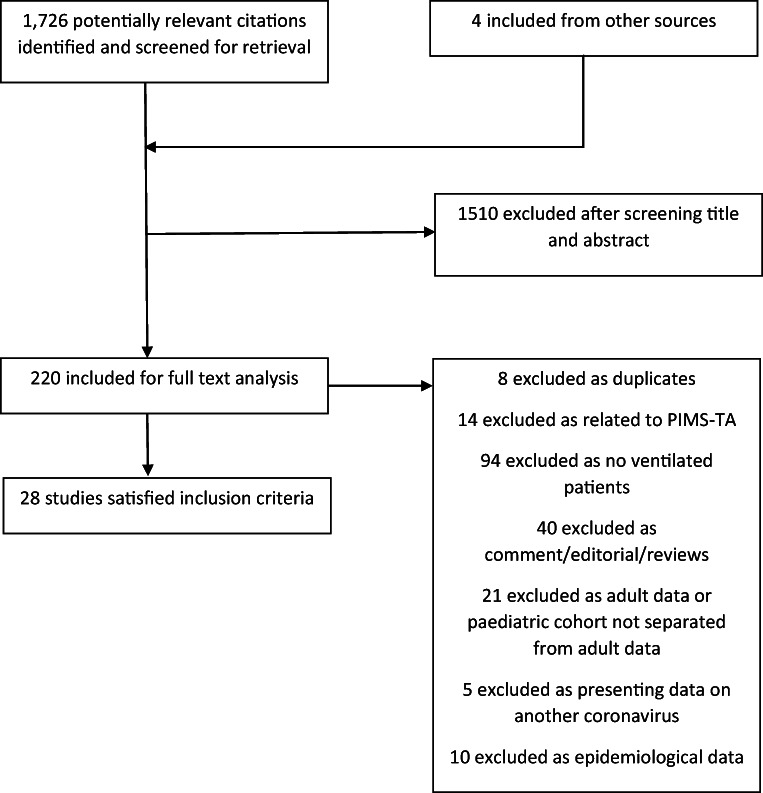
Quorum diagram

A total of 1,726 articles were retrieved from the electronic search and another 4 were included from a research bulletin recommended by an expert in the field. One thousand five hundred ten articles were excluded after previewing the title and abstract. The remaining 220 papers were retrieved for full text review. A total of 28 papers met the inclusion criteria and were included for analysis (Fig. 1).

We found a total of 5,686 paediatric cases of SARS-CoV, of which a total of 108 (1.9%) had severe SARS-CoV-2 requiring mechanical ventilation including 17 (0.3%) deaths. Not all studies reported details of patient comorbidities. Of the 108 children who were mechanically ventilated, the medical history was available for 48 patients. Thirty-six of the 48 (75%) had documented comorbidities and 12 (25%) were previously fit and well. Of the 17 patients who died, the past medical history was available for 12 patients; 8 had comorbidities (75%) and 4 (25%) did not (Table 1). One of the children who died was not intubated due to pre-existing comorbidities.

**Table 1:** Number of patients in included studies that required ventilation and/or died and their associated comorbidities

First author	Country	Ventilated patients *n*	Comorbidities in those ventilated *n* (%)	Details of comorbidities	Co-infection	Age of those mechanically ventilated
Cabrero-Hernandez [8]	Spain	1	0	NA		12 years
CDC [9]	USA	15	Not stated			Not stated
Chao [10]	USA	6	6 (100%)	Pt 1 metastatic cancer Pt 2 seizures, asthma Pt 3 congenital heart disease Pt 4 obesity Pt 5 seizures, quadreparesis Pt 6 HTN and OSA		Age of ventilated patients 4 months–15 years (mean 11.7 years)
Climent [11]	Spain	1	1 (100%)	Muccopolysacharidosis type 1		5 m
Cook [12]	UK	1	1 (100%)	Ex-preterm	Staph epidermis in blood culture	8 weeks
Coronado Munoz [13]	USA	1	0	NA		3 weeks
Craver [14]	USA	1	0	NA		17 years
DeBiasi [15]	USA	2	1 (50%)	Microcephaly, GDD, seizures gastrostomy		4 years and 16 years
Dong [16]	China	13	Not stated			
Garcia-Salido [17]	Spain	2	1 (50%)	Allogeneic hematopoietic stem cell transplantation		8 years and 12 years
Garazzino [18]	Italy	2	2 (100%)	Pt 1 ex preterm *n* = 1 (50%) Pt 2 congenital heart disease *n*		Neonate and 2 months old
Kanthimathinathan [19]	UK	1	1 (100%)	Congenital heart disease		Infant
Latimer [20]	USA	1	1 (100%)	18q deletion and epilepsy		18 years
Li [21]	China	1	1	Surgery for nephroblastoma	Mycoplasma	1 year
Lu [22]	China	3	3 (100%)	Pt 1 hydronephrosis Pt 2 leukaemia on maintenance chemotherapy Pt 3 intussusception		Not stated
Mannheim [23]	USA	7	4 (57%)	Cardiac/congenital heart disease *n* = 2 (50%) Chronic lung disease *n* = 1 (25%) Trisomy 21 *n* = 2 (50%) Immunodeficiency *n* = 1 (25%)		Infants represented 4 (40%) hospitalized children and 4 (57%) ICU patients
Oualha [24]	France	9	Not stated		One patient blood culture positive for Fusobacterium necrophorum and Strep. constellatus. One patient blood culture and CSF positive for Staph. aureus	
Parri [25]	Italy	1	1 (100%)	Epileptic encephalopathy		14 years 5 months
Patel [26]	USA	1	0	NA		12 years
Peng [27]	China	1	Not stated			Not stated
Qiu [28]	China	1	1 (100%)	Cardiac surgery and recurrent pneumonia		8 months
Shaw [29]	USA	1	1 (100%)	DORV D-malposed great arteries, subpulmonary ventricular septal defect (VSD), type A interrupted aortic arch, hypogammaglobulinemia immunodeficiency previous tracheostomy		3 years
Shekerdemian [30]	USA	18	Number of ventilated patients with comorbidities not stated (but 40/48 (83%) of patients admitted to PICU had underlying conditions)			
Tagarro [31]	Spain	1	Not stated			Not stated
Tullie [32]	UK	3	1 (33%)	Mild asthma		Children who were ventilated aged 8–14 (mean 11 years)
Wang [33]	China	3	Not stated	.		Not stated
Zachariah [34]	USA	9	8 (89%)	Obesity 6 (67%) Asthma 2 (22%) Immunosuppression 1 (11%) Neurological 1 (11%) Sickle cell disease 1 (11%) Cardiac 1 (11%) diabetes 2 (22%) genetic syndromes 2 (22%)		Infants less severely affected
Zheng [35]	China	2	2 (100%)	Pt 1 congenital heart diseases, malnutrition, and suspected hereditary metabolic diseases Pt 2 congenital heart disease	One patient positive for Enterobacter aerogenes	Both infant age group (8 and 11 months)

*NA* not applicable, *PICU* paediatric intensive care unit, *HTN* hypertension, *OSA* obstructive sleep apnoea, *GDD* global developmental delay, *ALL* acute lymphoblastic leukaemia

### Comorbidities

The details of documented comorbidities of those children who required mechanical ventilation with SARS-CoV-19 are summarised in Table 2.

**Table 2:** Documented comorbidities in mechanically ventilated children with SARS-CoV-19 (some patients had more than one comorbidity)

Cardiovascular	
Cardiovascular including congenital heart disease and cardiomyopathy	10/48 (21%)
Hypertension	1/48 (2%)
Mucopolysacharidosis with cardiac failure	1/48 (2%)
Neurological	
Epilepsy, neurodegenerative disorders and cerebral palsy	5/48 (10%)
Respiratory	
Asthma or reactive airway disease	5/48 (10%)
Recurrent chest infections	1/48 (2%)
OSA	1/48 (2%)
Immunosuppressed/Oncology/Haematology	
Allogeneic hematopoietic stem cell transplantation	1/48 (2%)
Leukaemia on maintenance chemotherapy	1/48 (2%)
Immunodeficiency	3/48 (6%)
Sickle cell disease	1/48 (2%)
Metastatic cancer	1/48 (2%)
Nephroblastoma	1/48 (2%)
Genetic syndromes	
Genetic syndrome unspecified	2/48 (4%)
T21	2/48 (4%)
18q deletion	1/48 (2%)
Endocrine	
Diabetes	2/48 (4%)
Obesity	7/48 (15%)
Other	
Prematurity	2/48 (4%)
Intussusception	1 (2%)
Hydronephrosis	1 (2%)
No comorbidity	12 (25%)

### Age

Many of the articles either did not report explicitly on the age of the patients with severe disease. For the patients who required mechanical ventilation or died for whom this data was available, 13 were < 1 year of age and 25 were > 1 year of age. Specifically, looking at the patients who died from SARS-CoV-19, age was documented in 13/17, of whom 2 were under the age of one (Table 3). The youngest patient to require mechanical ventilation was an ex preterm neonate in Italy and the youngest death reported was in a 5-month-old infant from Spain who had a history of muccopolysacharidosis type 1 and pre-existing cardiac failure.

**Table 3:** Demographics of patients who died with SARS-CoV-19

First author	Number who died	Age	Sex	Ethnicity	Comorbidities	Other details
CDC	3	–	–	–	–	–
Chao	1	11 y	M	Black	Metastatic cancer	Family chose to withdraw care after a period of invasive mechanical ventilation
Climent	1	5 m	M	–	Mucopolysaccharidosis with heart failure	Was on ACE inhibitor prior to admission
Craver	1	17 y	M	African American	Nil	Eosinophilic myocarditis on post mortem examination
Dong	1	14 y	M	–	–	–
Lu	1	10 m	–	–	Intussusception	
Oualha	5	16 y	F		Nil	
		16 y	M	–	Nil	Sphenoidal sinusitis with cavernous sinus thrombosis. Blood culture positive for Fusobacterium necrophorum and Strep. constellatus. Left middle cerebral artery stroke.
		6 y	F	–	Nil	Myocarditis and septic shock. Blood culture and CSF-positive for Staph aureus. Underwent ECMO and suffered massive brain haemorrhage.
		4 y	M	–	Chemotherapy for acute lymphoblastic leukaemia	ARDS and multiorgan failure
		17 y	F	–	Epilepsy and major neonatal encephalopathy	Not intubated due to mutual decision to withdraw care
Shekerdemian	2	12 y	–	–	Had comorbidities but no details given	Multiorgan failure
		17 y	–	–	Had comorbidities but no details given	Multiorgan failure
Wang	1	8 y	M	–	ALL in remission	
Zachariah	1	–	–	–	–	–

*M* male, *F* female, *y* year, *m* month

## Discussion

### Main findings

This is the first systematic review of children who have suffered critical illness following SARS-CoV-2 infection and in whom past medical history has been reported. In keeping with previous reports, the data presented show that the absolute risk of critical illness in children is low with intensive care treatment an infrequent occurrence. However, we have identified for the first time, that children with comorbidities have an increased relative risk of critical illness; this group comprising the majority of children who have required mechanical ventilation and the majority of children who have died. The comorbidities identified encompass a broad spectrum of diseases, cardiac disease being the most frequent. This is in keeping with a recent systematic review which looked specifically at cardiac disease in paediatric patients with SARS-CoV-19 and concluded that previous cardiac surgery is related with the risk of a more severe form of the disease [36].

### Interpretation

There are two fascinating features of SARS-CoV-2 as pertaining to disease in children; the risk of acquiring the infection appears to be lower than in adults (1% v 3.5%), and once infected, the risk of severe disease is almost 25 times lower than in adults [6]. The immune mechanisms underlying the duel phenomena of enhanced resistance to infection and enhanced resistance to severe disease are yet to be elucidated; however, the magnitude of this effect appears sufficient to protect most children with comorbidities from severe disease. Indeed, the data presented showed that only a small number of children with comorbidities actually suffered from critical illness, though data on pre-existing comorbidities was only available in 48 of the 108 patients who required mechanical ventilation.

Despite the low absolute risk of critical disease in children, the data presented show an increased relative risk for children with comorbidities. Chronic cardiac disease, respiratory disease and obesity are prominent comorbidities associated with critical disease. Interestingly, these comorbidities are also described as risk factors for severe disease in adults. In a large prospective observational cohort study of adults with severe COVID-19 infection, the most frequent comorbidities identified were chronic cardiac disease (29%), diabetes (19%), non-asthmatic chronic pulmonary disease (19%), asthma (14%) and obesity (11%) [37].

In contrast to adult data, immunological, haematological and oncological disease (with presumed immunosuppression) comprises 17% of comorbidities in the children described. This is surprising as it is thought that immunosuppression may have a protective effect in adults through interference with the aberrant inflammatory response associated with severe disease in adults [38]. Furthermore, studies of paediatric cohorts on immunosuppression have reported no increase in risk of severe disease [38–40]. Discerning an influence of immunosuppression may be difficult due to a relatively small effect, the influence of the underlying disease itself and the differing influences of different types of immunosuppression. Indeed, only a small number of immunosuppressed children identified in this study had critical disease implying that the absolute risk of critical disease associated with immunosuppression is small.

Older age has been found to be an important risk factor for severe disease in adults [37]. In children being less than 1 year of age has been reported to be a risk factor for severe disease [16]. In this review, we found that 35% of all children mechanically ventilated were infants under 1 year of age which suggests under 1’s are disproportionately affected by severe COVID-19. This is in keeping with a large European multi-centre study that found 29% of patients under 18 year of age infected with COVID-19 were in the infant age group and 48% of those admitted to ICU were under 2 years of age [41].

In the UK and the USA, countries with ethnically diverse populations, mortality is disproportionately high in adult populations of ethnic minority groups, and those of lower socio-economic status [42]. The complex factors underlying the relationship between COVID-19 and these demographic features are yet to be fully defined. We were unable to explore if these factors are important in severity of disease in children as socio-economic, and ethnicity data was rarely reported in the included studies.

The main weakness of this study was the potential missing data from studies that reported combined adult and paediatric data, where we were unable to extract the relevant paediatric data. Another weakness was that information on comorbidities was only available in 48 of the 108 patients who required mechanical ventilation, and more detail on the demographics and past medical history of all patients included would strengthen the conclusions and avoid selection bias. We are also aware that in this rapidly developing research area, new data is being published daily that may complement the data in this review. We must also stress that this data cannot be used to estimate individual risk as there is no universal testing; we cannot be sure how many children in a population are infected with COVID-19 at any one time. The majority of the included studies are from developed countries, and the impact on the developing world needs to be further studied. The key strength of this systematic review is that it is the largest study to date to look at the effect of comorbidities in children with severe COVID-19 and may be able to contribute to the discussion on social distancing and shielding in this population.

## Conclusion

Children with comorbidities have a predisposition to critical illness following infection with COVID-19 although the absolute risk remains low. These data are important in the assessment of risk with regard to the planned relaxation of social distancing measures for these children and their families. Prospective data collection is required to better define risk factors for severe disease including comorbidities, age, ethnicity and socio-economic status.

## Abbreviations

COVID-19: Coronavirus disease 2019
PIMS-TA: Paediatric multisystem inflammatory syndrome temporally associated with COVID-19
SARS-CoV-2: Severe acute respiratory syndrome coronavirus 2
